# Kidney Transplantation in Patients With aHUS: A Comparison of Eculizumab Prophylaxis Versus Rescue Therapy

**DOI:** 10.1097/TP.0000000000005135

**Published:** 2024-07-25

**Authors:** Caroline Duineveld, Emily K. Glover, Romy N. Bouwmeester, Nicole C.A.J. van de Kar, David Kavanagh, Jack F.M. Wetzels, Neil S. Sheerin

**Affiliations:** 1 Department of Nephrology, Radboud University Medical Center, Radboudumc Research Institute, Nijmegen, Gelderland, the Netherlands.; 2 National Renal Complement Therapeutics Centre, Newcastle Upon Tyne Hospitals NHS Foundation Trust, Newcastle Upon Tyne, United Kingdom.; 3 Department of Pediatric Nephrology, Amalia Children’s Hospital, Radboud University Medical Center, Radboudumc Research Institute, Nijmegen, Gelderland, the Netherlands.

## Abstract

**Background.:**

Guidelines advise eculizumab prophylaxis for most kidney transplant recipients with atypical hemolytic uremic syndrome (aHUS). However, recurrence rates may be overestimated, and starting eculizumab at relapse (“rescue therapy”) may prevent graft loss. Randomized controlled trials have not compared the efficacy, safety, and costs of different treatment strategies. We performed a comparative study, including a previously described Dutch cohort treated with rescue therapy and a UK cohort using eculizumab prophylaxis.

**Methods.:**

In the Netherlands, we selected all adult patients with aHUS who received a kidney transplant between 2010 and 2021 in the Radboud University Medical Center (n = 30) and enriched this cohort with 8 patients who received rescue therapy in other centers. The UK cohort included all adult patients with aHUS at moderate or high risk of recurrence, transplanted between 2013 and 2017 with prophylactic eculizumab.

**Results.:**

We included 38 Dutch patients and 35 UK patients. Characteristics were comparable, although the UK cohort included more patients with a complement factor H SCR20 mutation or hybrid gene (31% versus 5%; *P* < 0.01), and more Dutch patients received living donor kidneys (66% versus 20%; *P* < 0.001). Follow-up was comparable (the Dutch patients 70.8 mo, range, 10–134; UK patients 55.4 mo, range, 2–95). Eighteen (47%) Dutch patients received rescue therapy. Death-censored graft survival was not significantly different (the Dutch patients 1 y, 3 y, and 6 y: 97.4%, 91.2%, and 87.1%, respectively; UK patients 1 y, 3 y, and 6 y: 97.1%, 88.2%, and 65.6%, respectively, log-rank *P* = 0.189).

**Conclusions.:**

In a population characterized by low prevalence of “very high risk” genes, who were predominantly transplanted using an endothelial protective regime, death-censored graft survival with eculizumab rescue therapy was not inferior to prophylaxis.

## INTRODUCTION

Before the introduction of the C5 blocker eculizumab, the majority of patients with atypical hemolytic uremic syndrome (aHUS) developed end-stage renal disease.^1^ Kidney transplantation in patients with aHUS was associated with a high recurrence rate and limited graft survival.^2^ In 2016, the Kidney Disease: Improving Global Outcomes (KDIGO) consensus report advised prophylactic eculizumab therapy in aHUS patients at moderate or high risk of recurrence.^3^ Retrospective studies have shown that eculizumab prophylaxis is safe and prevents aHUS recurrence.^4,5^ The optimal duration of prophylaxis is debatable, as “late” aHUS recurrences, occurring >12 mo after transplantation, have been reported.^6^ Because of the high costs of eculizumab, the use of prophylactic therapy is not an option in all countries. Some centers have reported acceptable outcomes with eculizumab rescue therapy.^5-7^ No randomized controlled trials comparing prophylactic and rescue therapy have been performed. In the UK transplant recipients with aHUS, at moderate or high risk of recurrence, receive eculizumab prophylaxis with treatment continued indefinitely. In contrast, in the Netherlands transplant recipients with aHUS, irrespective of risk of recurrence, only receive eculizumab rescue therapy at the time of suspected aHUS recurrence after kidney transplantation.^8^ Recently, the outcome of kidney transplantation in patients with aHUS from the United Kingdom receiving eculizumab prophylaxis has been reported.^9^ Similarly, the Dutch CUREiHUS study reported the outcome of kidney transplantation using a strategy of eculizumab rescue therapy.^6^ In this retrospective comparative cohort study, we included adult patients from these cohorts and compared outcomes between eculizumab prophylaxis and eculizumab rescue therapy.

## MATERIALS AND METHODS

### Study Groups

In the Dutch cohort, all adult (older than 17 y) patients with aHUS (either with a history of aHUS before kidney transplantation or diagnosed with aHUS after transplantation) who received kidney transplantation between 2010 and 2021 in the Radboud University Medical Center (Radboudumc) were included. The outcome of this cohort has been described previously, we now provide longer follow-up.^6,8^ To enrich the cohort for complement factor H (CFH) mutations, 8 patients with aHUS, transplanted in another Dutch academic medical center, who received eculizumab rescue therapy and were included in the CURiHUS study, were added (**Figure S1**, **SDC**, http://links.lww.com/TP/D106).^6^ None of the patients received eculizumab prophylaxis. The patients from Radboudumc who were diagnosed with aHUS before transplantation preferentially receive a living donor kidney and are treated with an endothelial protective regime posttransplant.^³^ In short, the regime consists of induction therapy with basiliximab (20 mg on days 1 and 4), and triple therapy consisting of low-dose tacrolimus (starting dose, 0.03 mg/kg twice daily; target blood levels of 4–5 mg/L for the first 30 d, and 5–7 mg/L thereafter), prednisone (starting dose, 100 mg/d on days 1–3, thereafter 25 mg/d and tapering to 0.1 mg/kg/d at 3 mo after transplantation), and high-dose mycophenolate mofetil (starting dose, 1000 mg twice daily; target area under the curve, 40–60 mg/mL/h), strict blood pressure control (target, 130/80 mm Hg), and the early use of statins and angiotensin-converting enzyme inhibition.^8^ In the Radboudumc, this transplantation regime is used in all patients with aHUS, but in other Dutch academic centers, the transplantation regime used was at the discretion of the treating physician. Patients with aHUS are closely monitored after kidney transplantation. Details on the transplantation protocol and monitoring strategy are provided in **Supplemental Text S1** (**SDC**, http://links.lww.com/TP/D106).

In patients with a posttransplant aHUS, recurrence treatment with eculizumab was started according to the standard induction and maintenance scheme (induction phase: 900 mg at weeks 1, 2, 3, and 4; maintenance phase: 1200 mg starting at week 5, and then every 14 d 1200 mg). When measurement of free eculizumab levels became available, therapeutic drug monitoring was implemented and a weight-based induction scheme was introduced.^10,11^ Eculizumab withdrawal was considered after at least 3 mo of therapy, in patients fulfilling the following criteria: estimated glomerular filtration rate (eGFR) stable for at least 4 wk, no laboratory evidence of thrombotic microangiopathy (TMA; haptoglobin >0.3 mmol/L, platelet count >150 × 10^9^/L, lactate dehydrogenase <250 U/L) and no concurrent active triggers of endothelial injury (eg, calcineurin inhibitor toxicity, ongoing rejection, hypertension). The decision to withdraw therapy was made by the treating physician.

In the United Kingdom, all adult patients with aHUS (older than 17 y) at moderate or high risk of recurrence, who received a kidney transplantation between 2013 and 2017 with the use of eculizumab prophylaxis, were included. Patients received a single dose of 900 mg eculizumab before transplantation, 3 weekly doses of 900 mg after transplantation, continuing with maintenance therapy of 1200 mg biweekly thereafter. Eculizumab prophylaxis was intended to be continued indefinitely until graft loss or patient death.

In both countries, kidney biopsies were performed only on clinical indications.

### Data Collection

Data were extracted from medical notes and electronic patient records. Data on the first presentation of patients with aHUS in the native kidneys were collected, when available. Laboratory evidence of TMA (microangiopathic hemolytic anemia) was defined as at least 2 of the following 3 criteria: (1) thrombocytopenia (platelet count <150 × 10^9^/L, (2) lactate dehydrogenase greater than the upper limit of normal (>250 U/L), and (3) low haptoglobin (<0.3 mg/L). For Dutch patients, with a diagnosis of suspected recurrence of aHUS, biopsy reports were retrieved. Biopsies performed at the time of diagnosis of recurrence and start of eculizumab were evaluated for signs of active and chronic TMA and chronic injury. Biopsy-proven TMA was defined as arteriolar or glomerular thrombosis. To compare the difference in eculizumab use in both cohorts, the dosage of eculizumab administered was evaluated. In the Netherlands, the dosage of eculizumab (in grams) administered per patient was retrieved from electronic patient records. For the UK cohort, eculizumab dosage was calculated based on the standard dosing schedule (a single dose of 900 mg just before surgery, 3 weekly doses of 900 mg, then 1200 mg after a further week before continuing on 1200 mg every 2 wk). The outcome of the prophylactic and rescue treatment strategies was assessed by comparing aHUS recurrence rate, kidney function at 1 y after kidney transplantation, death-censored, and overall graft survival. A sensitivity analysis was performed in which Dutch patients who were diagnosed with aHUS after kidney transplantation were excluded.

### Genetic Analysis

In the Netherlands, complement genetic analysis was performed as part of routine work-up in clinical practice. In general, variant screening of CFH, complement factor I (CFI), complement factor B (CFB), complement C3 (C3), and membrane cofactor protein (MCP)/cluster of differentiation 46 and screening for chromosomal rearrangements using multiplex ligation-dependent probe amplification (MLPA) was performed. More recently, variant screening of complement factor 2 and complement factor properdine and noncomplement genes diacylglycerol kinase epsilon and thrombomodulin was added. In 15 patients, all transplanted before 2015, genetic screening was considered sufficient once a variant was found in 1 gene, and MLPA analysis was not done for all the patients (**Table S1**, **SDC**, http://links.lww.com/TP/D106). In the United Kingdom, MLPA analysis was performed for variant screening of CFH, CFI, CFB, C3, and MCP/cluster of differentiation 46. Sanger sequencing was performed to assess genetic abnormalities in noncomplement genes associated with aHUS including diacylglycerol kinase epsilon, metabolism of cobalamin associated C, vitronectin, plasminogen, thrombomodulin, and inverted formin 2, in selected patients. In both countries, no routine screening for factor H autoantibodies was undertaken. Genetic variants were classified according to the American College of Medical Genetics and Genomics guidelines.^12^

### Statistical Analysis

Group characteristics were compared using independent sample t-test or Mann-Whitney *U* test in case of continuous data and chi-square test or Fischer exact test in case of categorical variables. Renal graft survival was analyzed with Kaplan-Meier curves and was censored for patient death with a functioning graft (death-censored graft survival) and for a functioning graft at the last follow-up (overall graft survival). The Log-rank test assessed the difference between the survival of 2 groups. Analysis was performed using SPSS (version 25.0; IBM, Armonk, NY) and figures were drawn using GraphPad Prism (version 5.03, GraphPad Software, Boston, MA). A *P* value of <0.05 was considered statistically significant.

### Ethics Statement

In the Netherlands, the need for informed consent for the collection of retrospective and anonymized data from patients transplanted at Radboudumc were waived by the local medical ethics committee. Data collection for the CUREiHUS study was approved by the Medical Research Ethics Committee of Oost-Nederland (NL52817.091.15) in 2016. In the United Kingdom, the study was exempt from the National Health Service Research Ethics Review.

## RESULTS

The study included 38 adult Dutch patients (**Figure S1**, **SDC**, http://links.lww.com/TP/D106) and 35 adult UK patients. Six Dutch patients were diagnosed in retrospect with aHUS at the time of posttransplant recurrence, all other patients were known with aHUS before transplantation. Demographics, complement variants, and transplant details are provided in Table 1. Results of genetic analysis for individual Dutch and UK patients are provided in **Tables S1** and **S2** (**SDC**, http://links.lww.com/TP/D106). Most characteristics were comparable; however, several differences were noted. In the majority of the patients, a genetic variant was detected (mutational rate [VUS or pathogenic]: Dutch patients, 71% versus the UK patients, 80% [*P* = 0.376]). Although overall no difference was found in CFH, CFB, C3, and CFI variants between the 2 groups, the UK cohort includes more patients with hybrid genes and CFH mutations in SCR20 (respectively, 11/35 versus 2/38, *P* < 0.01). In both cohorts, most patients were classified as “high risk” for aHUS recurrence based on the classification recommended by KDIGO (the Dutch patients, 63% versus the UK patients, 71%).^3^ Dutch patients were more often transplanted with a living donor kidney, whereas patients from the United Kingdom more often received a deceased donor kidney (Dutch living donor, 66% versus the UK living donor, 20%; *P* < 0.001). The donors were of older age in the Netherlands (Dutch patients, 57 y, range, 23–74; UK patients, 49 y, range, 10–69; *P* = 0.037). All patients received a tacrolimus-based maintenance immunosuppressive regime. An endothelial protective transplantation regime (with low-dose tacrolimus) was used in 74% of Dutch patients.

**TABLE 1.** Patient demographics, complement variants, and transplant details

Variable	Dutch cohort, N = 38	UK cohort, N = 35	*P*
Female sex	29/38 (76.3%)	24/35 (68.6%)	0.459
Age at transplantation, y	45.4 (22.3–68.5)	42.0 (17.8–64.4)	0.101
History of aHUS	32/38 (87.9%)^*a*^	35/35 (100%)	0.026
Presentation in native kidneys with MAHA	29/33 (85.7%)	25/27 (92.6%)	0.681
Presentation in native kidneys with biopsy-proven TMA	15/16 (93.8%)^*b*^	22/22 (100%)	0.421
Presentation in native kidneys with AKI	33/33 (100%)	35/35 (100%)	ND
Genetic variant found (variant, n/patients, n)	35 variants/30 patients	30 variants/28 patients	ND
Type of variant (per total number of variants found)^*c*^	CFH 11/35 (31.4%) Hybrid gene 1/35 (2.9%) CFB 3/35 (8.6%) C3 18/35 (51.4%) CFI 2/35 (5.7%) MCP 0 (0%)	CFH 10/30 (33.3%) Hybrid gene 6/30 (20%) CFB 3/30 (10%) C3 9/30 (30%) CFI 2/30 (6.7%) MCP 0 (0%)	0.173
Classification of variant	(L)B 3/35 (8.6%) VUS 8/35 (22.9%) (L)P 24/35 (68.5%)	(L)B 0/30 (0%) VUS 7/30 (23.3%) (L)P 20/30 (76.7%)	0.347
Patients with variant in CFH SRC20 or hybrid gene	2/38 (5.3%)	11/35 (31.4%)	0.005
Previous KTx	13/38 (34.2%)	14/35 (40.0%)	0.609
Early aHUS recurrence in previous KTx	8/13 (61.5%)	11/14 (78.6%)	0.420
KDIGO recurrence risk	Moderate 14/38 (36.8%) High 24/38 (63.2%)	Moderate 10/35 (28.6%) High 25/35 (71.4%)	0.452
Type of kidney donor	Living 25/38 (65.8%) Deceased 13/38 (34.2%)	Living 7/35 (20%) Deceased 28/35 (80%)	<0.001
Living donor^*d*^	LRD 6/25 (24%) LURD 19/25 (76%)	LRD 5/7 (71.4%) LURD 2/7 (28.6%)	0.032
Deceased donor	DBD 12/13 (92.3%) DCD 1/13 (7.8%) (n = 13)	DBD 16/25 (64%) DCD 9/25 (36%) (n = 25)	0.118
Age donor (y)	57 (23–74) (n = 31)	49 (10–69) (n = 20)	0.037
Mismatch	3, 0–6 (n = 38)	3, 0–6 (n = 27)	0.615
Induction IS with basiliximab	35/38 (92.1%) (n = 38)	16/20 (80%) (n = 20)	0.219
Maintenance IS after discharge	TAC/MMF/prednisone 38^*e*^	TAC/MMF/prednisone 26 TAC/prednisone 2 TAC/MMF 1 TAC/AZA/prednisone 1 Unknown 5	ND

Results are given as n (%) or median (range). Data on presentation in native kidneys and transplant characteristics were unavailable for some patients.

^*a*^In 6 Dutch patients, the diagnosis of aHUS was made in retrospect after kidney transplantation.

^*b*^One Dutch patient in which the kidney biopsy showed severe vascular damage and global glomerulosclerosis (“end-stage kidney”).

^*c*^C3 R161W (c.481C>T) variant: Dutch cohort 13 of 18 (72.2%), UK cohort 1 of 9 (11.1%); CFH variants in SCR20: Dutch cohort 1 of 11 (9.0%), UK cohort 5 of 10 (50%).

^*d*^In **Supplemental Text S2** (**SDC**, http://links.lww.com/TP/D106), information regarding (genetic) evaluation of the living-related kidney donors can be found.

^*e*^Twenty-eight of 38 patients were treated with endothelial protective aHUS transplantation protocol.

aHUS, atypical hemolytic uremic syndrome; AKI, acute kidney injury; AZA, azathioprine; C3, complement C3; CFB, complement factor B; CFH, complement factor H; CFI, complement factor I; CNI, calcineurin inhibitor; DBD, donation after brain death; DCD, donation after circulatory death; IS, immunosuppression; KDIGO, Kidney Disease: Improving Global Outcomes; KTx, kidney transplantation; (L)B, (likely) benign; (L)P, (likely) pathogenic; LRD, living-related donor; LURD, living unrelated donor; MAHA, microangiopathic hemolytic anemia; MCP, membrane cofactor protein; MMF, mycophenolate mofetil; ND, not done; TAC, tacrolimus; TMA, thrombotic microangiopathy; VUS, variant of unknown significance.

The outcome after kidney transplantation is reported in Table 2. A recurrence was diagnosed in 10 patients of the Radboudumc cohort (recurrence rate 33%). The aHUS recurrence rate cannot be calculated for the other Dutch hospitals as the number of aHUS patients transplanted was unknown (**Figure S1**, **SDC**, http://links.lww.com/TP/D106). Eight patients transplanted in these hospitals were treated for posttransplant recurrence. Overall, the Dutch cohort included 18 patients (10 from the Radboudumc and 8 patients from other Dutch medical centers) with aHUS recurrence who received eculizumab “rescue” therapy. Recurrence was diagnosed at a median of 13.2 mo (range, 0.13–102.1) after transplantation. Half of the patients (9/18) presented with suspected recurrence >1 y after transplantation. Laboratory evidence of TMA was observed in 67% of patients (12/18), and a biopsy showed evidence of arteriolar or glomerular thrombosis in 85% (11/13). A detailed evaluation of these 13 biopsies is provided in **Supplemental Text S3** (**SDC**, http://links.lww.com/TP/D106). Two patients from the United Kingdom showed evidence of TMA on the kidney biopsy despite eculizumab prophylaxis. In 1 patient (No. 47), with delayed graft function, there were laboratory signs of TMA, and kidney biopsy performed on day 4 after transplantation showed active TMA. Terminal pathway activity was absent with ongoing eculizumab prophylaxis. The patient received plasma exchange, and repeated eculizumab dosing, and the graft continued to function. In another patient (No. 118), a biopsy, performed within 3 mo after transplantation, showed acute T cell–mediated rejection, with endothelial swelling and loss of fenestrations on electron microscopy, consistent with TMA. Subsequent biopsies did not demonstrate TMA until graft loss, 6 y posttransplant, when chronic TMA was identified.^9^ A biopsy-proven rejection was diagnosed in 13 (37%) UK patients and 13 (33%) Dutch patients (*P* = 0.673).

**TABLE 2.** Outcome after kidney transplantation

Variable	Dutch cohort n = 38	UK cohort n = 35	*P*
TMA (aHUS) recurrence	18/38 (47.4%)	2/35 (5.7%)^*a*^	0.001
Time to aHUS recurrence, mo	13.2 (0.13–102.1)	0.13 and 3	ND
Early aHUS recurrence (younger than 1 y)	9/18 (50%)	2/2 (100%)	ND
Systemic TMA at recurrence	12/18 (67%)	1/2 (50%)	ND
Biopsy-proven TMA at recurrence	Bx TMA 11 No TMA in Bx 2 No Bx done at the time of recurrence 4 Bx size too small 1	Bx TMA 1 No TMA in Bx 1	ND
Biopsy-proven rejection	13/38 (34.2%)	13/35 (37.1%)	0.793
sCreat at 1 y after KTx^*b*^, µmol/L	125 (59–485) (n = 37)	112 (57–518) (n = 28)	0.487
Last known status	Functioning 32 Graft loss 5 Death with functioning graft 1	Functioning 23 Graft loss 7 Death with functioning graft 5	0.107
Follow-up to last known status (m)	70.8 (9.6–133.7)	55.4 (1.9–95.4)	0.109
sCreat at last known status^*c*^, µmol/L	132 (59–284)	NA	
UPCR at last known status^*d*^ (g/10 mmol)	0.16 (0.05–1.95)	NA	
Graft loss cause	aHUS 3 Rejection 2^*e*^ Patient died 1	TMA 1 Sepsis 1 Rejection 4 Immune complex GN 1 Patient died 5	ND

Results are given as n (%) or median (range).

^*a*^Two patients developed TMA because they used eculizumab it is likely no aHUS recurrence but secondary TMA.

^*b*^Serum creatinine at 1 y after transplantation in patients with functioning grafts and available data. The Netherlands: 1 patient with graft loss <12 mo. United Kingdom: 2 patients with graft loss <12 mo, 5 missing values.

^*c*^Serum creatinine at last known status in patients with functioning grafts (n = 32).

^*d*^UPCR at last known status in patients with functioning grafts and available data (n = 29).

^*e*^Dutch cohort: 4 patients with graft loss were treated with eculizumab because of aHUS recurrence. In 1 patient, there was concurrent antibody-mediated rejection and graft loss occurred during ongoing eculizumab therapy. We consider the main reason for graft loss “rejection” in this patient.

aHUS, atypical hemolytic uremic syndrome; Bx, kidney biopsy; GN, glomerulonephritis; KTx, kidney transplantation; MMF, mycophenolate mofetil; NA, not available; ND, not done; sCreat, serum creatinine; TMA, thrombotic microangiopathy; UPCR; urine protein-creatinine ratio.

**FIGURE 1.**
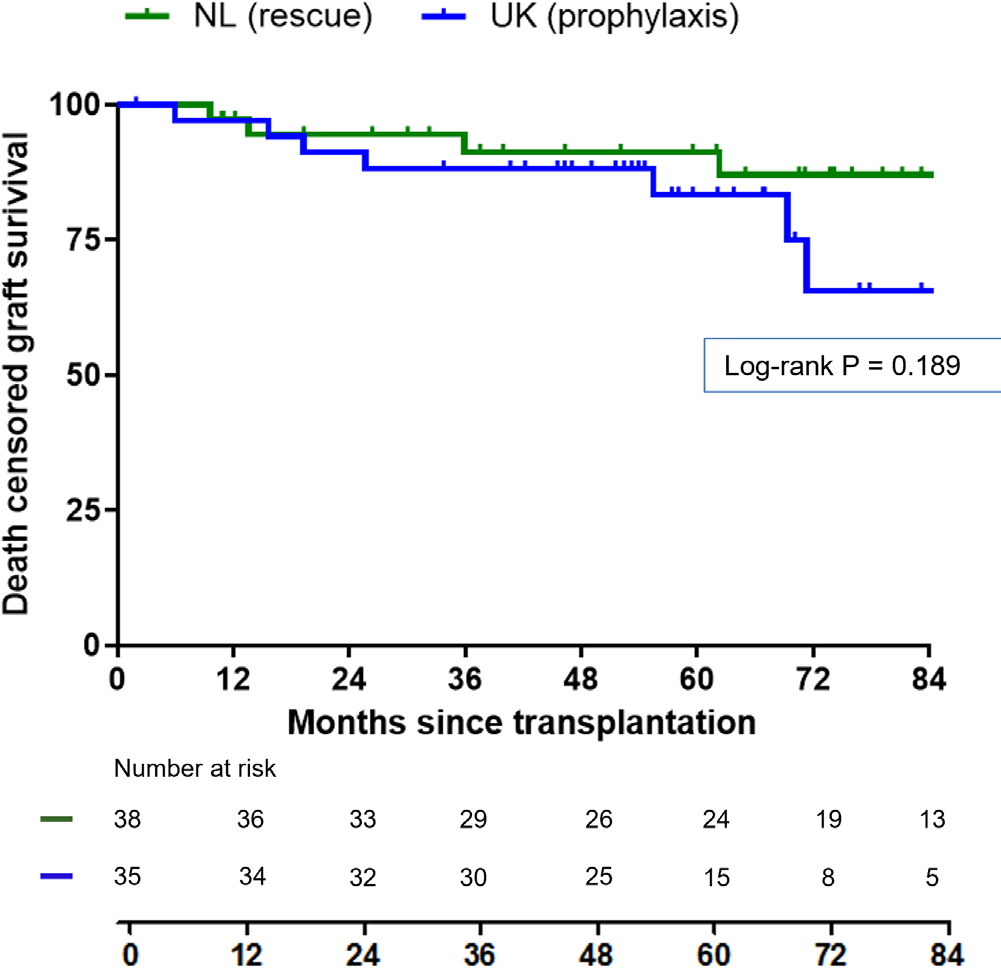
Death-censored graft survival. Kaplan-Meier analysis of death-censored renal graft survival in the Dutch cohort (the Netherlands; green), consisting of patients treated with a strategy of rescue therapy, and the UK cohort (blue), consisting of patients treated with a strategy of eculizumab prophylaxis. Numbers at risk in each group per 12-mo interval are indicated below the graph. Log-rank *P* = 0.189.

Based on an analysis of patients with functioning grafts and available data, serum creatinine at 1 y after transplantation was not significantly different between the 2 cohorts (Dutch patients, 125 µmol/L, range, 59–485 [n = 37] versus the UK patients, 112 µmol/L, range, 57–518 [n = 28]; *P* = 0.487). The follow-up to last known status was, respectively, 70.8 mo (range, 9.6–133.7) for the Dutch cohort and 55.4 mo (range, 1.9–95.4) for the UK cohort (*P* = 0.109). At the last follow-up, in the Dutch cohort, 32 grafts were still functioning, 5 grafts were lost (3 of them because of aHUS recurrence), and 1 patient had died with a functioning graft because of infectious complications combined with symptomatic aorta valve stenosis. In the UK cohort, 23 grafts were still functioning, 7 grafts were lost (1 to a TMA), and 5 patients had died with a functioning graft.^9^ The causes of death were sepsis (disseminated Candida and Herpes Simplex virus infection; n = 1), sudden death (n = 1), metastatic squamous cell cancer (n = 1), subarachnoid hemorrhage (n = 1), and myocardial infarction and adenocarcinoma (n = 1). No meningococcal infection-related deaths were seen. The deaths were deemed to be unrelated to eculizumab therapy. Although overall survival rate was better in the Dutch cohort (**Figure S2**, **SDC**, http://links.lww.com/TP/D106), death-censored graft survival was not significantly different between patients treated with rescue therapy and patients receiving eculizumab prophylaxis (the Netherlands, 1-y, 3-y, and 6-y death-censored graft survival of 97.4%, 91.2%, and 87.1%, respectively; the United Kingdom, 1-y, 3-y, and 6-y death-censored graft survival of 97.1%, 88.2%, and 65.6%, respectively, log-rank *P* = 0.189; Figure 1). A sensitivity analysis was performed in which Dutch patients who were diagnosed with aHUS after kidney transplantation (n = 6) were excluded, which did not affect results and conclusions (**Supplemental Text S4**, **SDC**, http://links.lww.com/TP/D106).

Nine Dutch patients were still being treated with eculizumab at the last follow-up (**Figure S3**, **SDC**, http://links.lww.com/TP/D106). Therapy had been discontinued in another 9 Dutch patients: in 1 patient because of nonresponse to eculizumab therapy, in 3 patients because of graft loss, and in the remaining 5 patients therapy was stopped after recovery or stabilization of kidney function and no laboratory signs of TMA. In 3 UK patients with a functioning graft, eculizumab prophylaxis was discontinued. Reasons to discontinue were declining kidney function, without evidence of TMA (n = 1), admission to intensive care unit (n = 1), and patient/physician preference (n = 1). In 2 patients, graft failure occurred after stopping prophylaxis however not due to aHUS.^9^

The total amount of eculizumab used in both strategies was evaluated. The Dutch cohort used 3796 mg eculizumab per patient-year versus 30 158 mg per patient-year in the UK cohort. Admittedly, the differences between the Dutch and UK scenarios will decrease with longer follow-up, especially when eculizumab therapy is continued lifelong in patients who start with rescue therapy. A sensitivity analysis, under the assumption that patients who have started eculizumab for rescue therapy would have continued its use, according to our therapeutic drug monitoring strategy, with no graft loss and a time horizon of 10 y, calculated 9712 mg eculizumab per patient per year in the Dutch versus 31 320 mg eculizumab per patient per year in the UK cohort (**Supplemental Text S5**, **SDC**, http://links.lww.com/TP/D106).

## DISCUSSION

The optimal treatment strategy for patients with aHUS after kidney transplantation is debated. The KDIGO consensus report suggested the use of eculizumab prophylaxis in all aHUS patients with an estimated moderate or high risk of disease recurrence.^3^ Although eculizumab prophylaxis prevents aHUS recurrence, such a strategy is associated with high costs. Furthermore, many patients do not develop a recurrence and thus are exposed unnecessarily to eculizumab. An alternative strategy is rescue treatment with eculizumab in case of posttransplant aHUS recurrence. In small studies, rescue treatment was effective, with TMA response and recovery of kidney function.^4,13-16^ Unfortunately, no randomized controlled trials have been performed. In the Netherlands, eculizumab treatment was introduced and reimbursed, under the condition of implementing a restrictive regime, limiting its use after kidney transplantation to patients with a diagnosis of aHUS recurrence. In contrast, in the United Kingdom, all aHUS patients at moderate or high risk of disease recurrence are given eculizumab prophylaxis. In this study, we evaluated the outcome of these strategies by comparing these previously reported cohorts from the United Kingdom and the Netherlands. The data indicate that rescue therapy was not inferior to prophylaxis with respect to overall and death-censored graft survival. In addition, less eculizumab was used in the Dutch cohort compared with the UK cohort. Although the differences in eculizumab use between the 2 cohorts will decrease with longer follow-up, especially when eculizumab therapy is continued lifelong in patients who start with rescue therapy, a sensitivity analysis with a 10-y horizon still showed major differences.

Few studies with a similar design have been performed. Si Nga et al^7^ reported low TMA-related graft loss rates in 10 aHUS patients treated prophylactically and 17 aHUS patients treated with rescue therapy (respectively, 10% and 5.9%), whereas the outcome was poor in untreated patients (graft loss rate attributed to TMA 91%). Superiority of eculizumab prophylaxis was suggested by Zuber et al^5^ who reported a significantly better death-censored graft survival in 52 patients receiving eculizumab prophylaxis compared with 29 patients who were treated with rescue therapy. However, in this study, 5-y graft survival was 84% in a subgroup of patients treated with rescue therapy, in whom eculizumab was started early (within 7 d) after diagnosis of recurrence. Based on an analysis of data from the Global aHUS Registry, Siedlecki et al^17^ found no significant difference in graft loss rates in 52 patients with aHUS treated with rescue therapy and 88 patients with aHUS treated prophylactically. The authors reported that patients treated with rescue therapy had a lower eGFR at 2 y after transplantation (respectively, 44.8 mL/min/1.73 m², range, 5–68; 70.2 mL/min/1.73 m², range, 49–83). Unfortunately, the data of the Global aHUS Registry are quite heterogeneous and insufficient to judge the appropriateness of diagnosis of aHUS recurrence and the interval until the start of eculizumab therapy.

In our comparative cohort study, graft survival was similar in the 2 cohorts, suggesting noninferiority of rescue therapy. Theoretically, we cannot exclude that there might be differences in markers of kidney injury. Unfortunately, data on eGFR, serum creatinine, or urine protein-creatinine ratio at the end of follow-up were not available for the UK cohort. Still, in the Dutch cohort there was no evidence of kidney injury at the end of follow-up (median serum creatinine 132 µmol/L [range, 59–284], and median urine protein-creatinine ratio 0.16 [range, 0.05–1.95]; Table 2). It is important to emphasize the need for close monitoring of kidney transplant recipients with aHUS who are not treated with prophylaxis. With strict monitoring early aHUS recurrences are likely detected early because of the frequent visits. However, late recurrences may be undetected for a longer period of time. As reported in our CUREiHUS study,^6^ only a small proportion of the patients with a late aHUS recurrence present after a trigger, manifesting TMA and a rapid decline in kidney function. Patients can be instructed to seek contact with their physician in case of potential triggers, to exclude aHUS recurrence. However, most patients with late recurrences presented with a slow loss of eGFR, without laboratory signs of TMA and in the absence of a trigger.^6^ In aHUS patients with a slow loss of kidney function a biopsy should be taken at an early stage to search for the cause of eGFR loss and if a TMA is present, eculizumab should be started. An analysis of kidney biopsies performed in Dutch aHUS patients with recurrence showed more chronic kidney injury (interstitial fibrosis, tubular atrophy, and glomerular sclerosis) in patients with late recurrence (>12 mo after transplantation), possibly reflecting late diagnosis.

Our study has limitations. The study is retrospective, and the cohorts were different in some aspects. The aHUS recurrence rate of 33% in our cohort from Radboudumc is relatively low compared with historical cohorts.^2,15,18,19^ This lower recurrence rate may be explained by the use of an endothelial protective transplant regime (which was not used in the patients diagnosed after transplantation with aHUS) or the preferred use of living donor kidneys. Also, in our cohort, few patients had variants in CFH or a hybrid gene. To better match the UK cohort, we therefore included all patients participating in the CURiHUS study, who were diagnosed with aHUS recurrence and received eculizumab rescue therapy. Herewith, the percentage of patients with aHUS recurrence increased to 47%, and the overall composition of the cohorts with respect to genetic variants became comparable. Still, the Dutch cohort clearly contained fewer patients with a variant in SCR20 of factor H or a hybrid gene. Differences in genetic variants are likely relevant because in a recent analysis of literature data mainly including patients with native kidney aHUS, the risk of recurrence was highest in patients with a factor H variant in SCR20.^20^ Likewise, one may argue that the C3 variant R161W, which was prevalent in our cohort, is a less pathogenic mutation. Functional data showed reduced binding of C3 to MCP (but normal regulation by CFH),^21^ classifying the variant as pathogenic and thus also as high risk according to the KDIGO.^³^ Timmermans et al^22^ reported 6 aHUS patients with an isolated C3 R161W variant, who received in total 10 kidney transplantations. The recurrence rate was 83% (5/6 patients, 7/10 transplants). Roumenina et al^21^ reported 4 aHUS patients with a C3 R161W variant, who received in total 7 kidney transplantations. Four grafts were lost caused by HUS recurrence. These data argue against the benign character of the variant.

The preferential use of living donor kidneys may also have contributed to the good outcome in our cohort. By using living donors ischemia reperfusion injury, an important cause of complement activation, is likely limited.^23^ However, Remuzzi et al^24^ also reported no aHUS recurrences in 7 patients transplanted with a deceased donor kidney. Notably, the authors used a similar endothelial protective transplant regime. When analyzing the outcome of our patients according to the type of donor, no significant differences were seen, arguing that the type of kidney donor may be less important (**Figure S4**, **SDC**, http://links.lww.com/TP/D106).

In conclusion, our study showed that, in regard to death-censored graft survival, rescue therapy is not inferior to eculizumab prophylaxis. Importantly, these conclusions only apply to patients similar to our Dutch cohort: treated with an endothelial protective transplant regime, with the preferential use of living donor kidneys, and including few patients with CFH variants in SCR20 or hybrid genes. Future studies should address the identification of very high-risk patients and should evaluate the benefits, risks, and costs of eculizumab prophylaxis versus rescue therapy. Such studies are needed in view of the healthcare costs that are increasing every year. Our data suggest that it is appropriate and ethically justified to develop randomized controlled trials.

## ACKNOWLEDGMENTS

The authors thank the following members of the Dutch atypical hemolytic uremic syndrome working group for their participation in this study: Dr S.P. Berger, Department of Nephrology, University Medical Center Groningen, Groningen; Dr M. Eijgelsheim, Department of Nephrology, University Medical Center Groningen, Groningen; Dr F.J. Bemelman, Department of Nephrology, Amsterdam University Medical Center, Amsterdam; Dr A. Nurmohamed, Department of Nephrology, Amsterdam University Medical Center, Amsterdam; and Dr J.W. van der Heijden, Department of Nephrology, Spaarne Gasthuis, Haarlem.

## Supplementary Material


