# Supplementary material for: Kidney Transplantation in Patients With aHUS: A Comparison of Eculizumab Prophylaxis Versus Rescue Therapy
Source: Transplantation. 2024 Jul 25;109(3):511–8. doi: 10.1097/TP.0000000000005135 (PMC11834958; doi:10.1097/TP.0000000000005135)
Supplement: Supplementary file 1 [file tpa-109-511-s001.pdf]

Table S1: genetic variants in individual Dutch patients

Patient number	Gene	Mutation	Mutation type#	Function (REF)	Population frequency	Pathogenicity	Previous transplant lost to aHUS^	KDIGO risk classification
1	CFH	c.1548T>A p.(Asn516Lys)	-	[3]	0.000405*	Benign		Moderate risk
2[1]	C3	c.481C>T p.(Arg161Trp)	-	[10]	0.0004	Pathogenic		High risk
	C3	c.3773A>C p.(Glu1258Ala)	-	NA	NA	Likely pathogenic		
3[2]	C3	c.481C>T p.(Arg161Trp)	-	[10]	0.0004	Pathogenic		High risk
4[2]	CFH	c.3638C>T p.(Arg1210Cys)	2 (C-terminal)	[4]	0.015	Pathogenic	Yes (<1 year)	High risk
5[1]	C3	c.193A>C p.(Lys65Gln)	-	[8]	0.0048	Pathogenic	Yes (<1 year)	High risk
6[2]	C3	c.481C>T p.(Arg161Trp)	-	[10]	0.0004	Pathogenic		High risk
7	Negative							Moderate risk
8	CFH	c.1520-1G>A	1	NA	0*	Pathogenic	Yes (<1 year)	High risk
9[3]	C3	c.481C>T p.(Arg161Trp)	-	[10]	0.0004	Pathogenic	Yes (late)	High risk
10[2]	C3	c.481C>T p.(Arg161Trp)	-	[10]	0.0004	Pathogenic		High risk
11	C3	c.26T>C p.(Leu9Pro)	-	NA	0.000547*	VUS	Yes (late)	Moderate risk
	CFH	c.388G>A p.(Asp130Asn)	-	[13]	0.000091*	VUS		
12	Negative						Yes (<1 year)	High risk

13	CFH	c.1423T>C p.(Tyr475His)	-	NA	NA	VUS		Moderate risk
14	C3	c.481C>T p.(Arg161Trp)	-	[10]	0.0004	Pathogenic	Yes (<1 year)	High risk
15[4]	C3	c.481C>T p.(Arg161Trp)	-	[10]	0.0004	Pathogenic		High risk
16[2]	Negative						Yes (late)	Moderate risk
17	CFH	c.2669G>T p.(Ser890Ile)	-	[14,15]	0.01994*	Benign		Moderate risk
18	Hybrid gene[6]	CFH/CFHR1 hybrid	FH hybrid	NA	NA	Pathogenic	Yes (<1 year)	High risk
19[2]	C3	c.481C>T p.(Arg161Trp)	-	[10]	0.0004	Pathogenic		High risk
20[2]	C3	c.481C>T p.(Arg161Trp)	-	[10]	0.0004	Pathogenic		High risk
21[2]	CFH	c.2850G>T p.(Gln950His)	-	[16]	0.003583*	VUS		Moderate risk
22	CFI	c.454G>A p.(Val152Met)	1	[12,17]	0.000025*	Pathogenic	Yes (<1 year)	High risk
23	Negative							Moderate risk
24	CFH	c.2034G>T p.(Trp678Cys)	1	NA	NA	Pathogenic	Yes (<1 year)	High risk
25	C3	c.481C>T p.(Arg161Trp)	-	[10]	0.0004	Pathogenic		High risk
26	C3	c.481C>T p.(Arg161Trp)	-	[10]	0.0004	Pathogenic		High risk
27	Negative							Moderate risk
28[2]	CFH	c.158G>A p.(Arg53His)	2 (N-terminal)	[18]	0*	Pathogenic		High risk
29	CFH	c.2872A>G p.(Lys958Glu)	-	NA	NA	VUS		Moderate risk
30	C3	c.1774C>T p.(Arg592Trp)	-	[7]	0.00004$	Pathogenic		High risk

	CFB	c.2024T>C p.(Val675Ala)	-	NA	0*	VUS		
31	Negative							Moderate risk
32[2]	CFB	c.1598A>G p.(Lys533Arg)	-	NA	0.01054	Benign		Moderate risk
33	Negative							Moderate risk
34	CFH	c.1520-1G>A	1	NA	0*	Pathogenic		High risk
	CFB	c.1697A>C p.(Glu566Ala)	-	NA	1.07	VUS		
35	C3	c.481C>T p.(Arg161Trp)	-	[10]	0.0004	Pathogenic		High risk
36	Negative							Moderate risk
37[2]	C3	c.481C>T p.(Arg161Trp)	-	[10]	0.0004	Pathogenic		High risk
38	C3	c.193A>C p.(Lys65Gln)	-	[8]	0.0048	Pathogenic		High risk
	CFI	c.1534+5G>T p.(IVS12+5)	-	NA	0.87	VUS		

Population frequency refers to that reported for each variant in the gnomAD database, * refers to the EXaC database. NA = population frequency not available

#: Type 1 (reduced cell surface expression) or Type 2 (normal expression with reduced function)

^ In patients with a late aHUS recurrence in previous transplant the diagnosis was made >1 year after transplantation.

[1] In these patients genetic screening of CFH, CFB, C3 and MLPA analysis was performed (screening of CFI and MCP was not performed).

[2] In these patients variant screening of CFH, CFI, CFB, C3 and MCP was performed (MLPA analysis was not performed).

[3] In this patient genetic screening of CFB, C3, and MLPA analysis was performed (screening of CFH, CFI, MCP was not performed).

[4] In this patient genetic screening of C3, CFI, CFH and MCP was performed (screening of CFB, and MLPA analysis was not performed).

Abbreviations: C3: complement C3, CFB: complement factor B, CFH: complement factor H, CFI: complement factor I, NA: not available, VUS: variant of unknown significance.

Table S2: genetic variants in individual UK patients

Patient number	Gene	Mutation	Mutation type#	Function (REF)	Population frequency	Pathogenicity	Previous transplant lost to aHUS^	KDIGO risk classification
47	CFH	c.2409C>A p.(Cys803*)	1	NA	0*	Pathogenic		High
52	CFH	c.2918G>A p.(Cys973Tyr)	1	[1]	0*	Pathogenic		High
56	CFH	c.3468dupA p.(Trp1157Metfs*22)	1	NA	NA	Pathogenic	Yes (<1 year)	High
60	CFH	c.3550A>G p.(Thr1184Ala)	2 (C-terminal)	[2,3]	0*	Pathogenic	Yes (<1 year)	High
64	CFH	c.3572C>T p.(Ser1191Leu)	2 (C-terminal)	[4]	0*	Pathogenic	Yes (late)	High
70	CFH	c.3643C>G p.(Arg1215Gly)	2 (C-terminal)	[4]	0.000008*	Pathogenic	Yes (<1 year)	High
85	Hybrid gene	CFH/CFHR1 hybrid	FH hybrid	[5]	NA	Pathogenic		High
93	CFH	c.3562_3564 deletion p.(Lys1188del)	2 (C-terminal)	NA	NA	Pathogenic		High
118	CFH	c.3643C>G p.(Arg1215Gly)	2 (C-terminal)	[4]	0.000008*	Pathogenic		High
138	Hybrid gene	CFH/CFHR1 hybrid	FH hybrid	[5]	NA	Pathogenic		High
166	CFH	c.1107G>A p.(Trp369*) from liver donor	1	[6]	0*	Pathogenic		High
176	Hybrid gene	CFH/CFHR1 hybrid	FH hybrid	[5]	NA	Pathogenic	Yes (<1 year)	High
177	Hybrid gene	CFH/CFHR1 hybrid	FH hybrid	[5]	NA	Pathogenic	Yes (<1 year)	High
179	Hybrid gene	CFH/CFHR1 hybrid	FH hybrid	[5]	NA	Pathogenic	Yes (<1 year)	High
264	C3	c.1775G>T p.(Arg592Leu)	-	[7]	0*	Pathogenic	Yes (late)	High
265	C3	c.193A>C p.(Lys65Gln)	-	[8]	0.0048	Pathogenic	Yes (<1 year)	High

267	C3	c.193A>C p.(Lys65Gln)	-	[8]	0.0048	Pathogenic		High
272	C3	c.3142C>G p.(Arg1042Gly)	-	[9]	0*	Pathogenic		High
278	C3	c.481C>T p.(Arg161Trp)	-	[10]	0.0004	Pathogenic	Yes (<1 year)	High
279	C3	c.485C>G p.(Thr162Arg)	-	NA	0*	VUS		Moderate
300	Negative							Moderate
314	CFH	c.2596+1G>C	1	NA	0.0004	Pathogenic		High
	CFI	c.355G>A p.(Gly119Arg)	1	[11]	0.042	Pathogenic		
318	Hybrid gene	CFH/CFHR1 hybrid	FH hybrid	[5]	NA	Pathogenic		High
383	C3	c.485C>G p.(Thr162Arg)	-	NA	0*	VUS		Moderate
387	C3	c.193A>C p.(Lys65Gln)	-	[8]	0.0048	Pathogenic		High
446	Negative							Moderate
554	Negative						Yes (<1 year)	High
557	CFB	c.1598A>G p.(Lys533Arg)	-	NA	0.01*	VUS		Moderate
558	Negative						Yes (<1 year)	High
559	CFI	c.1733T>C p.(Ile578Thr)	-	[12]	0.000025*	VUS	Yes (late)	Moderate
573	C3	c.3216G>T p.(Arg1072Arg)	-	-	NA	VUS	Yes (<1 year)	High
575	Negative							Moderate
578	CFB	c.724A>C p.(Ile242Leu)	-	-	0.00099*	VUS		Moderate
	CFB	c.1697A>C p.(Glu566Ala)	-	-	0.01107*	VUS		
581	Negative							Moderate

584	Negative							Moderate

Population frequency refers to that reported for each variant in the gnomAD database, * refers to the EXaC database. NA = population frequency not available

Type 1 (reduced cell surface expression) or Type 2 (normal expression with reduced function)

^ In patients with a late aHUS recurrence in previous transplant the diagnosis was made >1 year after transplantation.

Abbreviations: C3: complement C3, CFB: complement factor B, CFH: complement factor H, CFI: complement factor I, NA: not available, VUS: variant of unknown significance.

Figure S1: flow chart of Dutch cohort

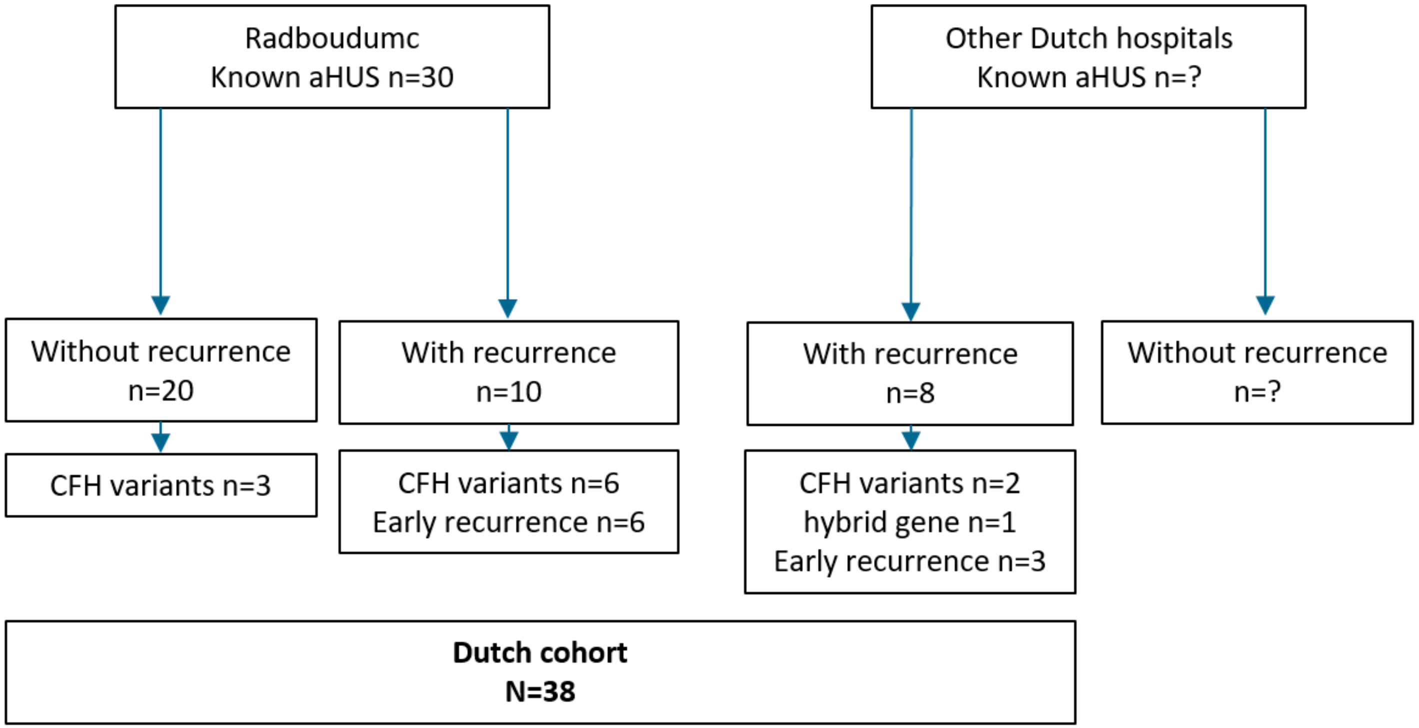

Legend: all patients (either known with aHUS before transplantation, or diagnosed with aHUS after transplantation) who received a kidney transplantation in the Radboud university medical center between 2010 and 2021 were included in the Dutch cohort. Recurrence rate in this cohort was 33% (10/30). Because of the relative low number of patients with CFH variants or hybrid genes 8 patients were added to make the Dutch cohort comparable to the UK cohort. These 8 patients, previously included in the CUREiHUS study, all received treatment with eculizumab because of posttransplant aHUS in another Dutch hospital.

<u>Figure S2: overall graft survival of Dutch and UK cohort</u>

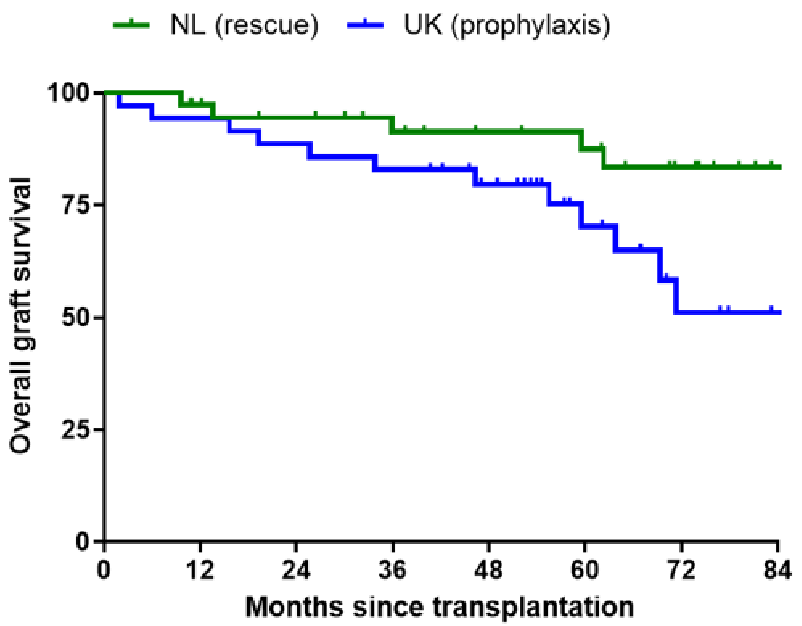

Number at risk

	0	12	24	36	48	60	72	84
NL (green)	38	36	33	29	26	24	19	13
UK (blue)	35	34	32	30	25	15	8	5

Legend: Kaplan-Meier analysis of overall renal graft survival in the Dutch cohort (NL) (green), consisting of patient treated with a strategy of rescue therapy, and the UK cohort (blue), consisting of patients treated with a strategy of eculizumab prophylaxis. Numbers at risk in each group per 12 month interval are indicated below the graph. Log-rank P = 0.026

<u>Figure S3: eculizumab therapy in Dutch patients with posttransplant recurrence (n=18)</u>

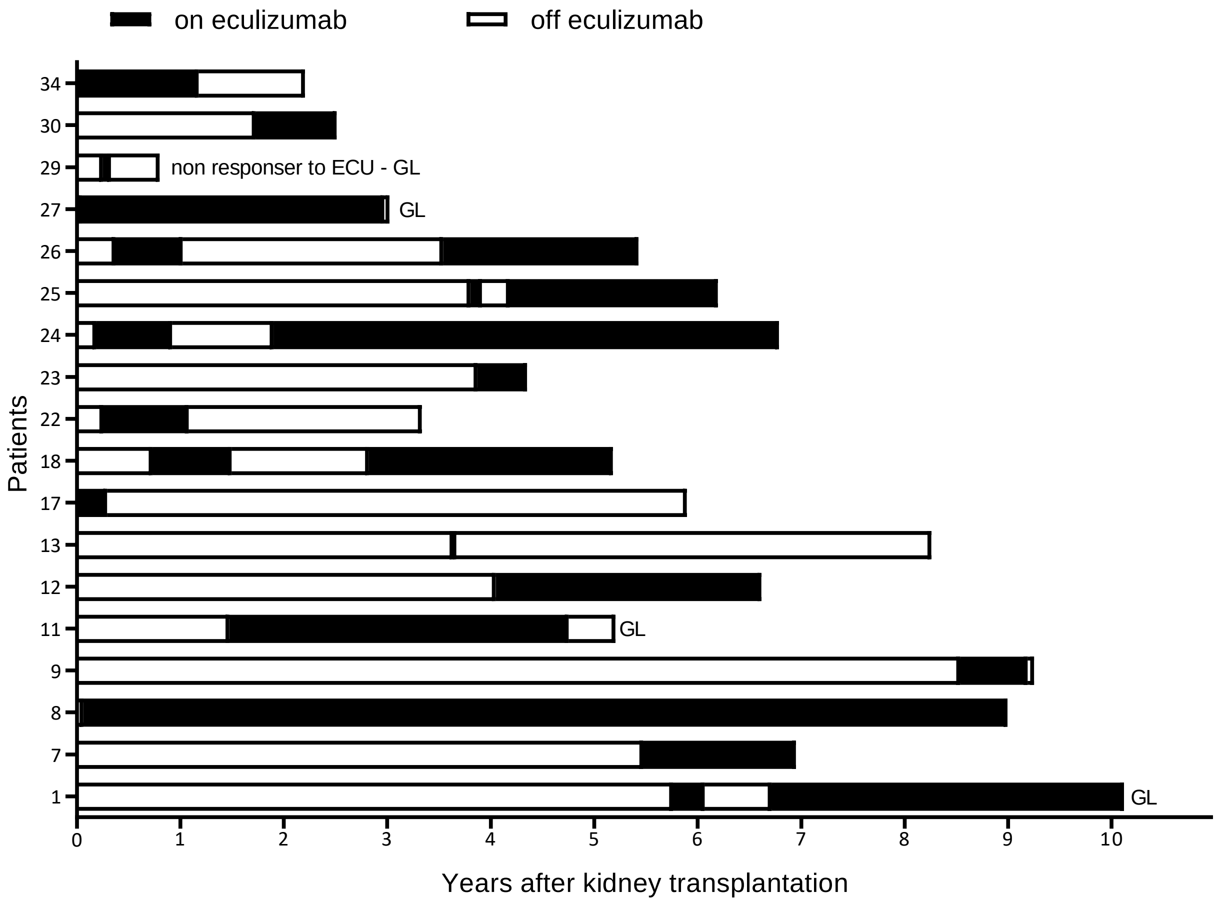

Legend: bar graph of 18 Dutch patients with posttransplant aHUS recurrence. The black bar illustrates time on eculizumab. The white bar illustrates time off eculizumab. GL: graft loss.

Figure S4: death censored graft survival per type of kidney donor

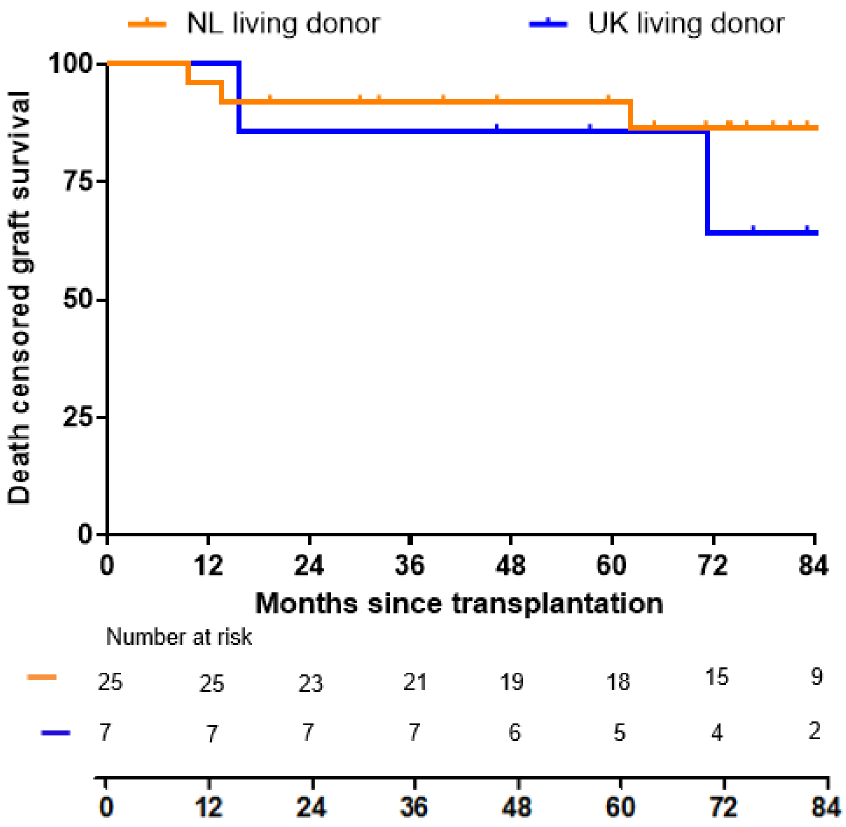

Legend: Kaplan-Meier analysis of death censored renal graft survival in the Dutch cohort (NL) (orange) and UK cohort (blue) for living donors. Numbers at risk in each group per 12 month interval are indicated below the graph. Log-rank P = 0.5748

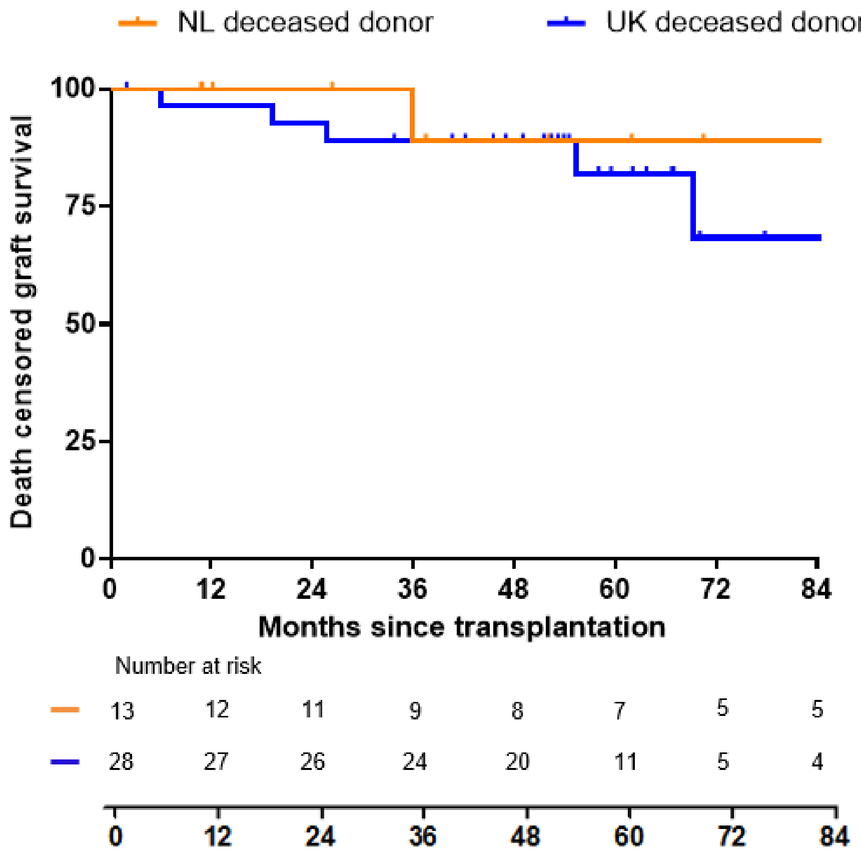

Legend: Kaplan-Meier analysis of death censored renal graft survival in the Dutch cohort (NL) (orange) and UK cohort (blue) for deceased donors. Numbers at risk in each group per 12 month interval are indicated below the graph. Log-rank P = 0.3750

Supplemental Text S1: Dutch aHUS transplantation protocol

In the Netherlands an endothelial protective transplantation protocol is recommended for aHUS patients. Atypical HUS patients are preferentially transplanted with a living donor kidney to minimize ischemia and reperfusion injury. In case there is no living donor, aHUS patients are transplanted with a donation after brain death (DBD) donor. Donation after circulatory death (DCD) donors are avoided. Family donors are genetically screened for complement variants according to guidelines.[19] Induction immunosuppressive therapy in aHUS patients is similar to patients with other primary kidney disease: basiliximab (20 mg on day 1 and day 4 after transplantation), or alemtuzumab in case of high sensibilized recipients (PRA >85%). After transplantation all patients are started on triple immunosuppressive therapy with tacrolimus, prednisolone and mycophenolate mofetil (MMF). The dosing schedule differs from what is used in non-aHUS patients: we aim for lower tacrolimus trough levels and higher MMF area under the curve (AUC) levels in aHUS patients. Tacrolimus is started at 0.03 mg/kg twice daily with target blood levels of 4-5 µg/L for the first 30 days, and 5-7 µg/L thereafter; prednisolone is started at 100 mg/d on days 1-3, thereafter 25 mg/d and tapering to 0.1 mg/kg/d at 3 months after transplantation; MMF is given at starting dose of 1,000 mg twice daily, with a target AUC of 40-60 mg/mL/h. The MMF AUC is usually measured at day 4-7 after transplantation, and this is repeated one or two times within 2-6 weeks after transplantation. After transplantation a blood pressure of <130/80 mmHg is targeted and an ACE inhibitor or angiotensin receptor blocker is preferably introduced in the first week after transplantation. Irrespective of cholesterol levels, a statin started directly after transplantation. Triple therapy is continued during the first year after transplantation. In the second year after transplantation triple therapy is preferentially continued, although the dosage of prednisolone may be lowered (and patients can be switched to double therapy on indication e.g. BKV infection, CNI toxicity, diabetes and obesity).

Patients with aHUS are closely monitored after kidney transplantation : daily for the first 2 weeks, twice weekly for the next 2 to 4 weeks, and weekly thereafter until 4 months after transplantation. Subsequently, the follow-up interval is gradually extended to every 6 weeks at 12 months and every 3 months after 2 years. Patients are instructed to perform home blood pressure measurements and antihypertensive medication is adjusted if required. Patients are also instructed to contact their physician in case of symptoms or signs compatible with aHUS recurrence (i.e., generalized malaise, high blood pressure, hematuria, edema, oliguria, dyspnea, and jaundice) or in case of potential triggers of aHUS (such as infections or vaccinations). At follow-up visits kidney function (creatinine and eGFR), hemolysis parameters (hemoglobin, thrombocytes, and lactate dehydrogenase [LDH]), and proteinuria were measured. Haptoglobin is measured in case of a suspected recurrence and infrequently during routine visits.

Supplemental Text S2: evaluation of living related kidney donors

In the Dutch cohort, 6 living related donors were used. In three patients aHUS was diagnosed after the kidney transplantation (see below), thus genetic evaluation of the donor was not performed. Of note, in 2 out of these 3 patients a genetic variant was found. Follow-up information is available for one donor: 7 years after donation kidney function is acceptable without proteinuria. In the three other patients aHUS was diagnosed before the kidney transplantation. All 3 patients carried a genetic variant. In two patients the donor was genetically screened before donation and no variant was found. In one patient no genetic tests were performed in the donor, however after 7 years of follow-up the donor has an acceptable kidney function without proteinuria. In the UK 5 patients received a living related donor. In all patients a genetic variant was found. In 3/5 the donor was screened and no genetic variant was found.

Table: genetic evaluation of donors in Dutch cohort

Case	Diagnosis aHUS	Genetic variant	Donor	Last FU donor
**1**	After Tx	CFH	Not tested (mother, 53y)	7y after donation: sCr 85 µmol/L, urine albumin 5 mg/ml
**29**	After Tx	CFH	Not tested (sister,57y)	Not available
**7**	After Tx	negative	Not tested (second cousin, age unknown)	Not available
**2**	Before Tx	C3	Not tested (mother, 63y)	7y after donation: sCr 97 µmol/L, eGFR (CKD-EPI) 51 ml/min/1.73m², urine albumin <3 mg/L
**4**	Before Tx	CFH	No mutation (mother, 58y)	
**19**	Before Tx	C3	No mutation (sister, 74y)	

Abbreviations: aHUS: atypical hemolytic uremic syndrome, CFH: complement factor H, C3: complement component 3, FU: follow-up, sCr: serum creatinine, Tx: transplantation, y:years.

Supplemental Text S3: evaluation of kidney biopsies of Dutch patients with a suspected posttransplant aHUS recurrence

We retrieved biopsy reports of patients with aHUS recurrence, with kidney biopsy done at the time of diagnosis of recurrence and start of eculizumab. Of 18 patients with aHUS recurrence, a biopsy was performed in 15 patients. We excluded one biopsy (from case 7) from analysis because it was taken 4.7 months before start of eculizumab. We also excluded one biopsy (from case 11) from analysis because of limited size (only 4 glomeruli). Of note, both excluded biopsies showed glomerular and/or arteriolar thrombi. In total, 13 biopsies could be evaluated. We compared the biopsy reports of patients with early (within 1 year after transplantation) versus patients with late (>1 year after transplantation) recurrence. Data are provided in the table.

In 8/9 patients with an early aHUS recurrence a kidney biopsy was performed. The biopsy was performed before start of eculizumab in 4 patients (maximal 10 days before), and the biopsy was performed after start of eculizumab in the remaining 4 patients (maximal 4 days thereafter). In all patients laboratory signs of TMA had been present before or at diagnosis of recurrence / start of eculizumab.

In 5/9 patients with a late aHUS recurrence a kidney biopsy was performed. In 4/5 patients the biopsy was performed before the start of eculizumab (maximal 15 days before). In 1/5 patients the biopsy was performed after start of eculizumab (4 days after). In 4/5 biopsied patients there had been no laboratory signs of TMA at all. In one patient there had been laboratory signs of TMA at start of eculizumab, but as the biopsy was taken after start of the C5 blocker, laboratory abnormalities has resolved at the time of biopsy.

More glomerular sclerosis and IFTA was seen in biopsies of patients with a late aHUS recurrence, compared to patients with an early recurrence. Signs of chronic TMA, such as double contour formation of the GMB, were also seen more often in patients with a late recurrence.

	**Early recurrence**	**Late recurrence**
	N=8	N=5
**Glomeruli (number (range))**	15 (7-45)	19 (13-60)
**Global sclerosis (% (range))**	0 (0-10%)	14 (0-50%)
**IFTA >25%**	0/8	3/5
**Intraglomerular thrombi**	+ 2/8<br>± 5/8	+ 2/5<br>± 1/5
**Intra-arteriolar thrombi**	+ 2/8<br>± 3/8	+ 1/5<br>± 1/5
**Double contour formations GBM**	+ 1/8<br>segmental 2/8	+ 4/5

Results are given as n (%) or median (range). ±: the biopsy reports of these patients “suggested” the presence of a thrombus in one segment, therefore we have classified these as ±.

There were 5 patients with evident active thrombi in glomeruli and/or arterioles (indicated as + in the table), of these, one patient never showed laboratory signs of TMA, and in two patients laboratory signs of TMA were not present at the time of biopsy.

The data is limited by the small number of patients.

Supplemental Text S4: sensitivity analysis excluding 6 Dutch patients without previous history of aHUS before kidney transplantation

There were 6 patients without a definite diagnosis of aHUS pretransplant. These 6 patients were all female, presented at relatively young age with ESRD (median 39y), and were diagnosed with "hypertensive nephropathy" as primary kidney disease. In 3 patients a genetic complement variant was found.. We have performed a sensitivity analysis, and exclusion of these 6 patients did not affect the results and conclusions, including death censored and overall graft survival (see below).

Sensitivity analysis - Table: Patient demographics, complement variants and transplant details

Variable	Dutch cohort n=32	UK cohort n=35	P value
Female gender	23/32 (71.9%)	24/35 (68.6%)	0.768
Age at transplantation (y)	46.3 (22.3-68.5)	42.0 (17.8-64.4)	0.092
Previous history of aHUS	32/32 (100%)	35/35 (100%)	ND
Presentation in native kidneys with MAHA	28/30 (93.3%)	25/27 (92.6%)	1.000
Presentation in native kidneys with biopsy proven TMA	14/14 (100%)	22/22 (100%)	ND
Presentation in native kidneys with AKI	32/32 (100%)	35/35 (100%)	ND
Genetic variant found (n variant/n patients)	32 variants / 27 pts	30 variants / 28 pts	ND
Type of variant (per total number of variants found) [1]	CFH 8/32 (25%)<br>Hybrid gene 1/31 (3.1%)<br>CFB 3/32 (9.4%)<br>C3 18/32 (56.3%)<br>CFI 2/32 (6.3%)<br>MCP 0 (0%)	CFH 10/30 (33.3%)<br>Hybrid gene 6/30 (20%)<br>CFB 3/30 (10%)<br>C3 9/30 (30%)<br>CFI 2/30 (6.7%)<br>MCP 0 (0%)	0.137
Classification of variant	(L)B 1/32 (3.1%)<br>VUS 7/32 (21.9%)<br>(L)P 24/32 (75%)	(L)B 0/30 (0%)<br>VUS 7/30 (23.3%)<br>(L)P 20/30 (76.7%)	1.000
Pts with variant in CFH SRC20 or hybrid gene	2/32 (6.3%)	11/35 (31.4%)	0.013
Previous KTx	12/32 (37.5%)	14/35 (40.0%)	0.834
Early aHUS recurrence in previous KTx	8/12 (67%)	11/14 (78.6%)	0.665
KDIGO recurrence risk	Moderate 8/32 (25%)<br>High 24/32 (75%)	Moderate 10/35 (28.6%)<br>High 25/35 (71.4%)	0.742
Type of kidney donor	Living 22/32 (68.8%)<br>Deceased 10/32 (31.3%)	Living 7/35 (20%)<br>Deceased 28/35 (80%)	<0.001
Living donor	Living related 3/22<br>Living unrelated 19/22	Living related 5/7<br>Living unrelated 2/7	0.008
Deceased donor	DBD 10/10 (100%)<br>DCD 0/10 (0%)<br>(n=13)	DBD 16/25 (64%)<br>DCD 9/25 (36%)<br>(n=25)	0.036
Age donor (y)	57 (23-74) (n=27)	49 (10-69) (n=20)	0.076
Mismatch	3, 0-6 (n=31)	3, 0-6 (n=27)	0.488

Induction IS with basiliximab	30/32 (93.8%)<br>(n=38)	16/20 (80%)<br>(n=20)	0.189
Maintenance IS after discharge	TAC/MMF/pred 32/32 (100%) [2]	TAC/MMF/pred 26<br>TAC/pred 2<br>TAC/MMF 1<br>TAC/AZA/pred 1<br>Unknown 5	ND

Results are given as n (%) or median (range). Data on presentation in native kidneys and transplant characteristics were unavailable for some patients.

[1] C3 R161W (c.481C>T) variant: Dutch cohort 13/18 (72.2%), UK cohort 1/9 (11.1%); CFH variants in SCR 20: Dutch cohort 1/8 (12.5%), UK cohort 5/10 (50%).

[2] 28/32 patients were treated with endothelial protective aHUS transplantation protocol.

Abbreviations: aHUS: atypical hemolytic uremic syndrome, AKI: acute kidney injury, AZA: azathioprine, CNI's: calcineurin inhibitors, DBD: donation after brain death, DCD: donation after circulatory death, IS: immunosuppression, n.a.: not applicable, KTx: kidney transplantation, (L)B: (likely) benign, (L)P: (likely) pathogenic, LRD: living related donor, LURD: living unrelated donor, MAHA: microangiopathic hemolytic anemia, MMF: mycophenolate mofetil, ND: not done, pts: patients, TAC: tacrolimus, TMA: thrombotic microangiopathy, y: years

Sensitivity analysis excluding 6 Dutch patients without previous history of aHUS before kidney transplantation - Table: outcome after kidney transplantation

Variable	Dutch cohort n=32	UK cohort n=35	P value
aHUS recurrence	12/32 (37.5%)	2/35 (5.7%) [1]	0.003
Time to aHUS recurrence (months)	13.2 (0.26-102.1)	0.13 and 3	ND
Early aHUS recurrence (<1y)	6/12 (50%)	2/2 (100%)	ND
Systemic TMA at recurrence	8/12 (67%)	1/2 (50%)	ND
Biopsy-proven TMA at recurrence	Bx TMA 6<br>No TMA in Bx 2<br>No Bx done at time of recurrence 3<br>Bx size too small 1	Bx TMA 1<br>No Bx TMA 1	ND
Biopsy-proven rejection	10/32 (31.3%)	13/35 (37.1%)	0.612
sCreat at 1 year after KTx [2] (µmol/L)	122 (59-485) (n=32)	112 (57-518) (n=28)	0.756
Last known status	Functioning 29<br>Graft loss 2<br>Death with functioning graft 1	Functioning 23<br>Graft loss 7<br>Death with functioning graft 5	0.055
FU to last known status (m)	72.4 (10.8-133.7)	55.4 (1.9-95.4)	0.100
sCreat at last known status [3] (µmol/L)	130 (59-267)	n.a.	
UPCR at last known status [4] (g/10mmol)	0.16 (0.05-1.1)	n.a.	
Graft loss cause	aHUS 1<br>Rejection 1<br>Patient died 1	TMA 1<br>Sepsis 1<br>Rejection 4<br>Immune complex GN 1<br>Patient died 5	ND

Results are given as n (%) or median (range).

[1] two patients developed TMA, since they used eculizumab it is likely no aHUS recurrence but secondary TMA.

[2] serum creatinine at 1-y after transplantation in patients with functioning grafts and available data. UK: 2 pts with graft loss <12 months; 5 missing values.

[3] serum creatinine at last known status in patients with functioning grafts (n=29).

[4] UPCR at last known status in patients with functioning grafts and available data (n=29).

Abbreviations: aHUS: atypical hemolytic uremic syndrome, FU: follow-up, GN: glomerulonephritis, n.a.: not available, KTx: kidney transplantation, m: months, MMF: mycophenolate mofetil, ND: not done, sCreat: serum creatinine, TMA: thrombotic microangiopathy, UPCR; urine protein-creatinine ratio, y: year.

Sensitivity analysis excluding 6 Dutch patients without previous history of aHUS before kidney transplantation - Figure: death-censored graft survival

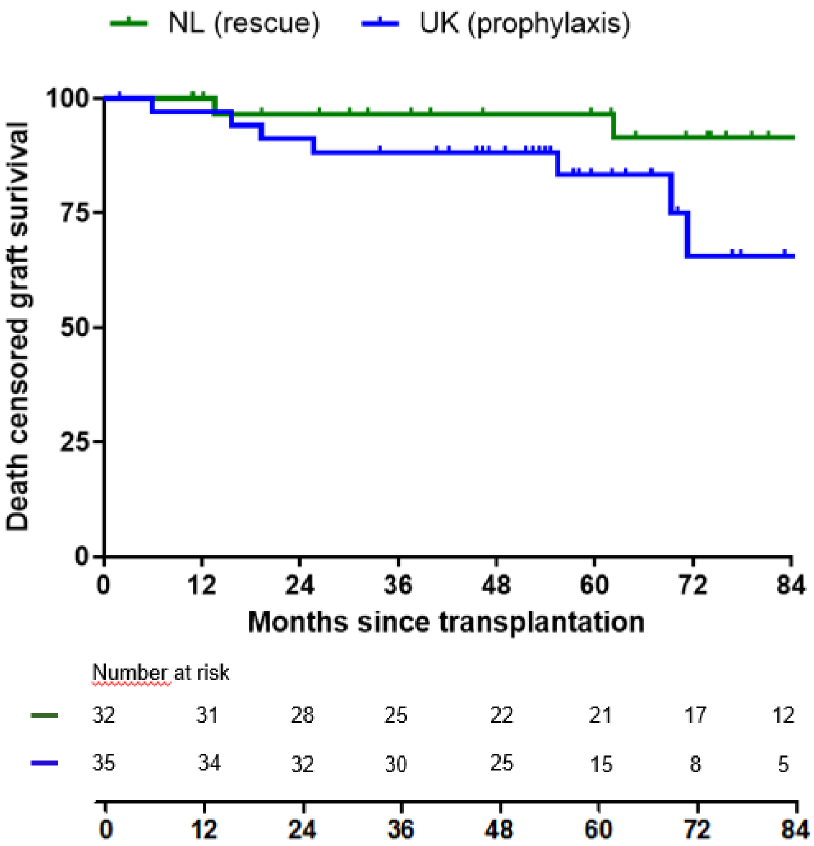

Legend: Kaplan-Meier analysis of death-censored renal graft survival in the Dutch cohort (NL) (green), consisting of patient treated with a strategy of rescue therapy (n=32), and the UK cohort (blue), consisting of patients treated with a strategy of eculizumab prophylaxis. Numbers at risk in each group per 12 month interval are indicated below the graph. Log-rank P = 0.061

Sensitivity analysis excluding 6 Dutch patients without previous history of aHUS before kidney transplantation - Figure: overall graft survival

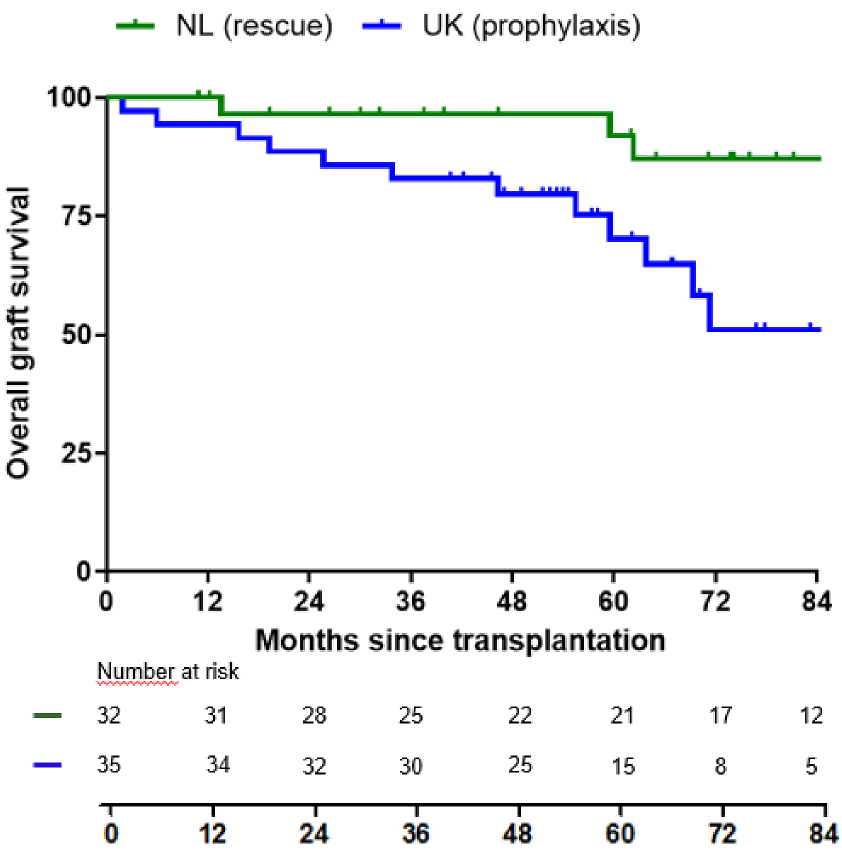

Legend: Kaplan-Meier analysis of overall renal graft survival in the Dutch cohort (NL) (green), consisting of patient treated with a strategy of rescue therapy (n=32), and the UK cohort (blue), consisting of patients treated with a strategy of eculizumab prophylaxis. Numbers at risk in each group per 12 month interval are indicated below the graph. Log-rank P = 0.010

Supplemental Text S5: eculizumab use in the Dutch and UK cohort

The total amount of eculizumab used in the Dutch and UK strategy was evaluated. In the Netherlands 18 patients were treated with eculizumab. Treatment was individualized used TDM. In total 810,000 mg eculizumab was administered. The cumulative follow-up duration of these 18 patients was 113 years, thus 7,197 mg eculizumab was used per patient per year. When evaluating the entire Dutch cohort 810,000 mg eculizumab was used in 38 patients, with a cumulative follow-up duration of 213 years, thus 3,796 mg eculizumab was used per patient per year. For the UK cohort the standard dosing schedule (a single dose of 900 mg just before surgery, 3 weekly doses of 900 mg, then 1200 mg after a further week before continuing on 1200 mg every 2 weeks) was used to calculate the dose of eculizumab used. In total 4,872,365 mg eculizumab was used. The cumulative follow-up duration of these 35 patients was 162 years, thus 30,158 mg eculizumab was used per patient per year.

Admittedly, the differences between the Dutch and UK scenario's will decrease with longer follow-up, especially when eculizumab therapy is continued lifelong in patients who start with rescue therapy. Therefore, we have performed a sensitivity analysis, under the assumption that patients who have started eculizumab for rescue therapy would have continued its use, according our TDM strategy, with no graft loss and a time horizon of 10 years, and with censoring of patients who have not started eculizumab at the time of last follow-up (to exclude any bias from patients with very late recurrence). The yearly dose of eculizumab under a TDM strategy is 20,004 mg. Based on our cohort of 38 patients, with start of rescue therapy in 18, and median time from transplantation to start of therapy of 13.2 months (range 0.13-102.1), we calculated a cumulative dose of 2,845,564 mg of eculizumab, and a total follow-up of 293 years, which amounts 9,712 mg of eculizumab per patient per year. If the patients in the UK cohort would all have continued eculizumab until 10 years after kidney transplantation, and no graft losses would have occurred, 10,962,000 mg of eculizumab would have been used. The cumulative follow-up would have been 350 years (10y * 35 pts), thus 31,320 mg eculizumab would have been used per patient per year.

References supplemental appendix

1. Saland, J.M., et al., *Favorable long-term outcome after liver-kidney transplant for recurrent hemolytic uremic syndrome associated with a factor H mutation.* Am J Transplant, 2006. **6**(8): p. 1948-52.
2. Wilson, C., et al., *Successful simultaneous liver-kidney transplant in an adult with atypical hemolytic uremic syndrome associated with a mutation in complement factor H.* Am J Kidney Dis, 2011. **58**(1): p. 109-12.
3. Martín Merinero, H., et al., *Functional characterization of 105 factor H variants associated with aHUS: lessons for variant classification.* Blood, 2021. **138**(22): p. 2185-2201.
4. Kavanagh, D., T.H. Goodship, and A. Richards, *Atypical hemolytic uremic syndrome.* Semin Nephrol, 2013. **33**(6): p. 508-30.
5. Nester, C.M., et al., *Atypical aHUS: State of the art.* Mol Immunol, 2015. **67**(1): p. 31-42.
6. Brown, J.H., et al., *Postpartum aHUS secondary to a genetic abnormality in factor H acquired through liver transplantation.* Am J Transplant, 2012. **12**(6): p. 1632-6.
7. Fremeaux-Bacchi, V., et al., *Mutations in complement C3 predispose to development of atypical hemolytic uremic syndrome.* Blood, 2008. **112**(13): p. 4948-52.
8. Volokhina, E., et al., *Novel C3 mutation p.Lys65Gln in aHUS affects complement factor H binding.* Pediatr Nephrol, 2012. **27**(9): p. 1519-24.
9. Schramm, E.C., et al., *Mapping interactions between complement C3 and regulators using mutations in atypical hemolytic uremic syndrome.* Blood, 2015. **125**(15): p. 2359-69.
10. Roumenina, L.T., et al., *A prevalent C3 mutation in aHUS patients causes a direct C3 convertase gain of function.* Blood, 2012. **119**(18): p. 4182-91.
11. van de Ven, J.P., et al., *A functional variant in the CFI gene confers a high risk of age-related macular degeneration.* Nat Genet, 2013. **45**(7): p. 813-7.
12. de Jong, S., et al., *Effect of rare coding variants in the CFI gene on Factor I expression levels.* Hum Mol Genet, 2020. **29**(14): p. 2313-2324.
13. Wong, E.K.S., et al., *Functional Characterization of Rare Genetic Variants in the N-Terminus of Complement Factor H in aHUS, C3G, and AMD.* Front Immunol, 2020. **11**: p. 602284.
14. Biggs, R.M., et al., *An Evaluation of the Complement-Regulating Activities of Human Complement Factor H (FH) Variants Associated With Age-Related Macular Degeneration.* Invest Ophthalmol Vis Sci, 2022. **63**(12): p. 30.
15. Tortajada, A., et al., *Complement factor H variants I890 and L1007 while commonly associated with atypical hemolytic uremic syndrome are polymorphisms with no functional significance.* Kidney Int, 2012. **81**(1): p. 56-63.
16. Mohlin, F.C., et al., *Functional characterization of two novel non-synonymous alterations in CD46 and a Q950H change in factor H found in atypical hemolytic uremic syndrome patients.* Mol Immunol, 2015. **65**(2): p. 367-76.
17. Java, A., et al., *Functional Analysis of Rare Genetic Variants in Complement Factor I (CFI) using a Serum-Based Assay in Advanced Age-related Macular Degeneration.* Transl Vis Sci Technol, 2020. **9**(9): p. 37.
18. Pechtl, I.C., et al., *Disease-associated N-terminal complement factor H mutations perturb cofactor and decay-accelerating activities.* J Biol Chem, 2011. **286**(13): p. 11082-90.
19. Goodship, T.H., et al., *Atypical hemolytic uremic syndrome and C3 glomerulopathy: conclusions from a "Kidney Disease: Improving Global Outcomes" (KDIGO) Controversies Conference.* Kidney Int, 2017. **91**(3): p. 539-551.
